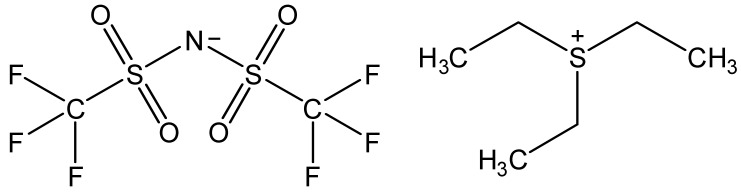# Protein and Polysaccharide-Based Fiber Materials Generated from Ionic Liquids: A Review

**DOI:** 10.3390/molecules25153362

**Published:** 2020-07-24

**Authors:** Christopher R. Gough, Ashley Rivera-Galletti, Darrel A. Cowan, David Salas-de la Cruz, Xiao Hu

**Affiliations:** 1 Department of Physics and Astronomy, Rowan University, Glassboro, NJ 08028, USA; goughc2@students.rowan.edu (C.R.G.); riveragaa4@students.rowan.edu (A.R.-G.); cowand35_@students.rowan.edu (D.A.C.); 2 Department of Chemistry and Biochemistry, Rowan University, Glassboro, NJ 08028, USA; 3 Department of Chemistry, and Center for Computational and Integrative Biology, Camden, NJ 08102, USA; ds1191@camden.rutgers.edu; 4 Department of Biomedical Engineering, Rowan University, Glassboro, NJ 08028, USA; 5 Department of Molecular and Cellular Biosciences, Rowan University, Glassboro, NJ 08028, USA

**Keywords:** protein, polysaccharides, fibers, biomaterials fabrication, tissue engineering, drug delivery, ionic liquid, filtration, green solvents

## Abstract

Natural biomacromolecules such as structural proteins and polysaccharides are composed of the basic building blocks of life: amino acids and carbohydrates. Understanding their molecular structure, self-assembly and interaction in solvents such as ionic liquids (ILs) is critical for unleashing a flora of new materials, revolutionizing the way we fabricate multi-structural and multi-functional systems with tunable physicochemical properties. Ionic liquids are superior to organic solvents because they do not produce unwanted by-products and are considered green substitutes because of their reusability. In addition, they will significantly improve the miscibility of biopolymers with other materials while maintaining the mechanical properties of the biopolymer in the final product. Understanding and controlling the physicochemical properties of biopolymers in ionic liquids matrices will be crucial for progress leading to the ability to fabricate robust multi-level structural 1D fiber materials. It will also help to predict the relationship between fiber conformation and protein secondary structures or carbohydrate crystallinity, thus creating potential applications for cell growth signaling, ionic conductivity, liquid diffusion and thermal conductivity, and several applications in biomedicine and environmental science. This will also enable the regeneration of biopolymer composite fiber materials with useful functionalities and customizable options critical for additive manufacturing. The specific capabilities of these fiber materials have been shown to vary based on their fabrication methods including electrospinning and post-treatments. This review serves to provide basic knowledge of these commonly utilized protein and polysaccharide biopolymers and their fiber fabrication methods from various ionic liquids, as well as the effect of post-treatments on these fiber materials and their applications in biomedical and pharmaceutical research, wound healing, environmental filters and sustainable and green chemistry research.

## 1. Introduction

Biomaterials derived from natural products have been of interest in recent decades due to their abundance, low cost, biocompatibility and tunable morphological and physical properties [1,2,3,4,5]. These materials have been broadly used for the development of membranes and fibers for liquid and gas separation [6] and sensing [7,8], fabrication of tissue engineering scaffolds for neural [9] or bone regeneration [10] and for the fabrication of nanostructures for drug delivery [11]. The study of biomaterials includes aspects of medicine, biology, chemistry, engineering, environmental science and materials science. These materials are extremely versatile as shown from a variety of biopolymers used in nature, such as in spider silk which has the overall highest tensile strength in nature while maintaining a very high elasticity, and chitin that is used by many insects to provide structural stability and protection. However, to make biopolymers largely available in modern technology, there needs to be the development of new methodologies to tune the properties of these materials to suit specific technological demands.

Protein and polysaccharide polymers are composed from the basic building block of life: amino acids and carbohydrates. Understanding the rule of life of these two systems is critical to unleash a flora of new materials that could revolutionize the way we fabricate multi-structural and multi-functional systems. Natural proteins and polysaccharides are relatively inexpensive and easy to process and regenerate, making it an attractive material from an economic perspective. The main advantage of these biopolymer-based materials over synthetic materials in the biomedical field is their unique biodegradability and biocompatibility. Minimizing the host immune response is an important aspect of determining the success of a drug delivery or tissue regeneration operation. Protein and polysaccharide-based biomaterials play a critical role in this process since they can be degraded by natural enzymes in the body, which reduces the accumulation of harmful by-products [12,13]. 

Solvents are essential, as they are the driving force for the dissolution of the protein and the polysaccharide. A poor solvent will significantly affect the miscibility of protein or polysaccharide polymers and reduce the mechanical properties of the final materials [14,15,16]. In addition, many organic solvents will markedly alter the original molecular weight of protein materials such as silks [17]. Therefore, the use of ionic liquid (IL) is frequently applied for tuning the final properties of the material. Ionic liquids have been used to dissolve both polysaccharides and selected proteins such as silk, keratin and collagen without changing their molecular weights [18,19,20]. They have an advantage over organic solvents as they do not lead to unwanted side products and are considered as a green alternative as they encompass the ability to be reusable [21]. The composition of an ionic liquid is very important as it combines a bulky asymmetric cation with a weakly coordinated anion, which can cause changes in the intra- and inter-molecular interactions [22,23,24,25]. When biopolymers are dissolved in ionic liquids, the anion forms hydrogen bonds with hydroxyl groups in the biopolymer, disrupting the naturally occurring hydrogen bonding network while the cation associates with the ether oxygen atoms and CH groups [26], thus causing a change in the molecular conformation. To improve interactions of a biopolymer material, such as silk or keratin between itself or other materials, one can increase interfacial adhesion while mitigating interfacial tension of the material. This can be accomplished by dissolving the biomacromolecules and reforming them in a solvent, via a coagulation process, to promote the stabilization and formation of secondary structures embedded in a matrix by hydrogen bonds, electrostatic interactions and covalent bonds [23,24,26]. In addition, the coagulation solvent could act as a nucleating agent to increase the nucleation and overall crystallization rate leading to changes in the natural conformation of the biopolymers [27]. Controlling the formation of protein secondary structures and polysaccharide crystallites is crucial for these studies.

The use of fibrous materials in biomedical research is becoming increasingly popular due to their high surface-area-to-volume ratio, mechanical strength, porosity and tunability. Fibers made from natural biopolymers are biodegradable and biocompatible and therefore enzymes in the body can degrade these fibers into nontoxic metabolites that can be reabsorbed. The incorporation of natural biopolymers into biological materials has also been shown to enhance cell attachment due to the presence of native cell attachment motifs and increase cell migration and proliferation [28,29]. Therefore, the use of biopolymer-based fibers in tissue engineering and nanomedicine has medical and commercial appeal. However, despite these advantages, the improvement of the mechanical and physical properties of biopolymer-based fibers remains challenging. Thus, biopolymer-based fibrous materials can be further modified by crosslinking or blending with other biocompatible materials to form a tunable platform. These characteristics are also highly dependable on the type of manufacturing method used to make the fibers.

This review provides a basic knowledge of the commonly used materials and methods for the fabrication of protein or polysaccharide-based fibers through various ionic liquids, as well as their corresponding use in tissue engineering, water filtration and drug delivery. Popular proteins and polysaccharides such as keratin, collagen, silk, elastin, corn zein, soy, cellulose and chitin are of particular interest. Electrospinning is currently the most popular fabrication technique, with several modifications also used, such as wet/dry jet spinning. Protein and polysaccharide materials have also been shown to vary depending on the solvent used (ionic liquid type or other organic solvents), the fiber post-treatment (such as various coagulation agents), as well as the composition of the fiber. In addition, this review article will discuss the current understanding of interactions between natural polysaccharides and structural proteins through their composite materials, which directly affect the morphology and physical, thermal and mechanical properties of the matrix in ionic liquids, such as the material crystallite formation and protein secondary structure in 1D fibers.

## 2. Typical Biopolymers from Proteins and Polysaccharides

**Figure 1:**
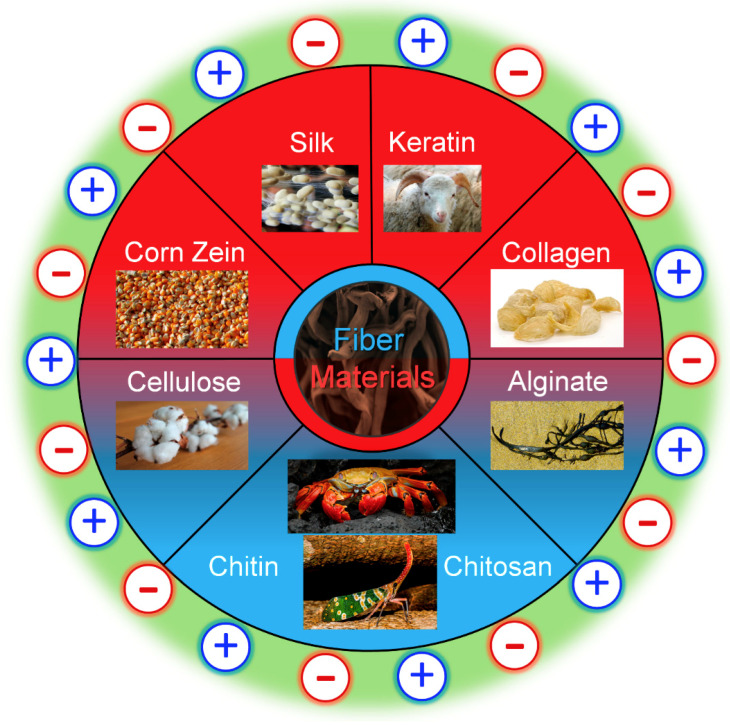
Commonly used protein and polysaccharide fiber materials that can be generated from ionic liquids with anions and cations and their natural sources. Protein materials are in red, while polysaccharides are in blue.

Several distinct kinds of proteins and polysaccharides are used in present-day research, including composite mixtures of both. Some commonly used materials are listed below in Figure 1 with their sources. Depending on the desired application and properties of the material being made, however, other proteins and polysaccharides can be used. The main difference between proteins and polysaccharides is that proteins are composed of amino acids, while polysaccharides are composed of long chains of sugar molecules. On a smaller scale, proteins are synthesized from long chains of peptides to form their primary structure. From there, various interactions with itself or other proteins including hydrogen and disulfide bonding, London dispersion forces and charge–charge interactions allow proteins to form higher-order structures including coils, helices and sheets. Polysaccharide chains tend to be more limited in their interactions, which limits their higher-order structures. Nonetheless, polysaccharides are long chains of sugar monomers that interact with each other and other polysaccharides through glycosidic linkages and hydrogen bonding to form polymers of long chains. In addition, many hybrid materials are made that combine proteins and polysaccharides together.

### 2.1. Protein Materials

#### 2.1.1. Silk Proteins

Silk protein, generally, is a fibrous material that is spun by a variety of arthropod species. These fibers are semi-crystalline with an ordered structure within a nebulous matrix [30]. Five different structures have been identified in silk proteins with vastly different molecular organization including coiled coil, extended beta sheet, cross-beta sheet, collagen-like triple helix and polyglycine II [31,32,33]. These unique structures require specific amino acid sequences in the proteins, and each provide unique material properties to the protein. This means different silk proteins vary in their mechanical strength, thermal properties, bioactivity, chemical activity and how well they blend into composites with other proteins. Due to this, silk has been used as thin protein films [29,34,35] and nanofibers [36,37,38] in a wide variety of applications including delivery of drugs [39] and cytokines [40], tissue engineering [38,41,42] and textile electronics [43,44,45]. Silk has been used in the textile industry for hundreds of years and is mechanically strong. The ability to tune its mechanical properties by varying the beta sheet content makes silk a widely used biomaterial today. Plentiful literature has shown the ability of silk to dissolve in ionic liquids, including for the formation of fiber materials [18,46,47,48,49].

#### 2.1.2. Keratin Proteins

Keratin is another naturally derived protein being used for biomedical and bioelectronic purposes. Keratin is often extracted from wool fibers and processed into nanofibers [50,51]. Other sources of keratin include feathers, hair fibers, fingernails and horns. Depending on the source, different forms of keratin can be extracted. α-keratin, for example, creates fibrils made up of intermediate filaments. Although keratin is mechanically weaker than silk, studies have been conducted to try to improve its mechanical strength [52]. Like other biomaterials, keratin is often used for its high biocompatibility and strong material properties. The applications of keratin include tissue engineering [53,54], drug delivery [55] and food packaging [56]. Several different types of keratin exist in nature, due to an abundance of amino acid substitutions that occur in the molecule. For example, keratin 14 is a structural protein found in basal cells in the epithelium when paired to keratin 5; when the methionine amino acid in keratin 14, a hydrophobic, nonpolar amino acid, is substituted with threonine, a hydrophilic, polar amino acid, a severe skin disease is developed [57]. Other mutations can also cause less severe diseases, such as substituting methionine to arginine in the linker region of the keratin. The unique amino acids in keratin also serve a strong purpose in packaging; the high amount of polar amino acids in natural keratin sources allows for membranes with high adsorption for Cu(II) and other metals [50]. Due to the strong outer cuticle in keratin fibers due to heavy crosslinking with disulphide bonds, many common solvents are unable to dissolve it. Ionic liquids, however, show potential as a solvent for dissolving keratin due to the ability of select ILs to disrupt disulphide bonds [58,59]. This ability gives ILs strong potential in keratin fiber formation.

#### 2.1.3. Soy Proteins

Another attractive protein for material applications is soy extracts. Soy proteins are globular proteins consisting of two main units, conglycinin 7S and glycinin 11S. Both of these units are primarily dominated by a random coil structure and contain subproteins of varying molecular weights. They can be either water-soluble albumins or globulins soluble in salt solutions, with the majority of soy proteins being the latter [60]. Isolates from soy protein contain several functional groups that can interact with the surrounding environment, making them useful for several applications like pollutant filtering [61], adhesives [60] and inhibiting oxidation [62]. In general applications, soy proteins are often used in tissue engineering [63,64] and have been used to create microcapsules [65]. Since soy proteins and isolates from soy proteins lack the mechanical integrity of other proteins, researchers often create composites with more mechanically stable polymers in order to create strong biomaterials with the application potential of soy. Due to this, the cosolvent potential of ILs is highly appealing and widely studied [66,67].

#### 2.1.4. Collagen

Collagen can assemble into 29 different types, each with its own type distribution and signaling abilities in vivo [68], but the majority of collagen in the body is either collagen I, II or III. Collagen’s repeating amino acid sequence allows it to form a stable secondary protein structure consisting of triple helices. These helices can further assemble into quaternary structures, which allows collagen to assemble into fibrillar proteins found throughout the body and in collagen V and XI. Upon dissolution in IL, the triple helical structure of collagen can be partially destroyed, but still regenerate into fiber [69]. Additionally, collagen is dissolvable in several types of IL [70] with both stabilizing or destabilizing effects depending on the ionic effects of the IL [71].

Nanofibers, protein films and hydrogels are all commonly used forms of collagen in material engineering. Type I collagen is the most widely used form of collagen as a biomaterial due to its abundance in nature. It also allows for successful mimicry of the human extracellular matrix (ECM), since collagen I is a major component of it.

#### 2.1.5. Elastin

Elastin is a vital protein found in the ECM of the body in order to provide elasticity and resiliency to tissues and organs. Where collagen provides structure and strength to the body, elastin provides flexibility. Elastin is primarily found in the lungs, aorta and skin, where elasticity is crucial [72]. During fiber formation, elastin molecules bind to other ECM proteins and uncoil into elongated chains which are able to crosslink with each other by oxidizing lysine residues [73]. Due to its elasticity and origins near the ECM, elastin is a popular biomaterial choice for vascular tissue engineering and other materials that need to interface with blood [74]. Elastin is also frequently used as a skin substitute to treat burns and chronic wounds due to its importance in native skin tissue, however the actual elastin content is low in these scaffolds [75]. Elastin scaffolds must be combined with a mechanically stronger material such as collagen. Self-assembling proteins are a popular topic in current research and this topic area is where elastin receives frequent attention. Currently, there is literature on elastin’s ability to self-assemble in fibers, hydrogels [76], sheets [77], nanoparticles [78], sponges [79] and nanoporous materials [80]. Although elastin is difficult to dissolve, IL can be used to separate it from biomass [81].

#### 2.1.6. Corn Zein

Zein is a major storage protein of corn, making it a very abundant source for biomaterials and it is also easily extracted. As a coproduct of the bioethanol industry, zein is abundant. Corn zein comes in several different forms. As a nanofiber, it tends to show a random coil protein structure, and as a thin film, it tends to show a more alpha-helical protein structure. It is widely used as a biomaterial due to its abundance and ease to extract, while maintaining good biocompatibility. In current research, zein is frequently used in tissue engineering [82,83] and drug delivery [84]. Zein can also be chemically modified in order to improve its mechanical properties [85]. ILs have proven to be an effective solvent for zein proteins, obtaining a concentration of up to 10% *w*/*w* in BMIMCl and BMIMdca or up to 15% wt% in C_4_C_1_ImCl [86]. The highest concentration found in the literature was 70 wt% from the protic ILs NH_3_(CH_2_CH_2_OH)OFo and NH_3_(CH_2_CH_2_OH)OAc [87], although this produced impractical, viscous solutions. In the same study, more practical solutions of 20 wt% were produced with conventional heating at 120 °C or using a microwave.

#### 2.1.7. Reflectin

A relatively new class of proteins, first reported in 2004, is the reflectin protein. These proteins are given their name from their unique spectral and optical properties, which allow them to self-assemble into reflecting materials [88]. In nature, reflectin proteins have been identified in cephalopods and in squids, where both animals use reflectin’s optical properties for camouflage. The dynamic structural colors caused by this protein are reported to be due to hierarchical assembly at the nanoscale assembly level [89]. Better understanding of this assembly could lead to the design of biomaterials with complex optical functions such as contact lenses. Recently, reflectin was used for a neural stem cell scaffold in order to promote human neural stem/progenitor cell function [90]. Being a new material, the solubility of reflectin in IL has not directly been studied; however, reflectin has been dissolved in organic solvents with the intent to process it into fibers [91]. Research has shown that “osmotic motors” control the refractive index of reflectin proteins, with positively charged linker segments that are restricted by Coulombic repulsion [92]. ILs, then, could potentially use ionic interactions to tune the refractive properties of reflectin for specific applications. By neutralizing the linker segments in reflectin, these linker segments are able to overcome Coulombic repulsion and re-assemble into multimeric spheres of well-defined size and dispersity [93]. The wide range of anions available in ILs could result in several different self-assembly positions for reflectin for a wide variety of applications.

### 2.2. Polysaccharide Materials

#### 2.2.1. Starch

Starch is cited as one of the most promising materials for biodegradable films due to its abundance, low cost, biodegradability and renewability [94]. The molecular structure of starch is made up of two main structures. Polysaccharide amylose forms linear structures in starch while amylopectin forms branched structures. The amount of each of these varies with the source of the starch, but ranges from 20–25% amylose and 75–80% amylopectin, generally. While amylose is semi-crystalline and soluble in hot water, amylopectin is very crystalline and does not dissolve in water. Chemically, amylose is connected by α-1,4 linked glucose units while amylopectin is branched by short α-1,4 chains linked by α-1,6 bonds [95]. On the macroscale, starch is highly hydrophilic. Due to this, it is disadvantaged in its brittleness, poor elasticity and poor water resistance. To overcome this, starch is often mixed with fillers that work to minimize these weaknesses. Starch is chemically connected as a series of anhydrous glucose units connected by primarily α-d-(1 → 4) glucosidic bonds [95]. ILs have been used to dissolve starch both by itself in concentrations up to 10% *w*/*w* [86], and with other biopolymers such as cellulose to form fibers [96].

#### 2.2.2. Cellulose

Cellulose is one of the most ubiquitous natural polysaccharides. Numerous sources of cellulose exist in nature, including trees, plants and fruits, due to its important role in the cell wall in plants. Some strains of bacteria are also able to synthesize cellulose. The molecular structure of cellulose consists of repeating glucopyranose molecules covalently linked through acetal functions between hydroxyl groups. It is a linear homopolysaccharide with several hydroxyl groups in the thermodynamically favorable position. During synthesis, cellulose forms microfibrils (2–30 nm diameter) with both crystalline and amorphous regions. Microfibrils will aggregate into bigger fibrils (30–100 nm diameter, 100–500 μm length) and then into fibers (100–400 nm diameter, 0.5–4.0 mm length) [97]. Several studies have proven the effectiveness of IL as a solvent for cellulose [98,99,100,101] and cellulose composites [48,49,102], making it one of the most promising materials for the fabrication of fiber materials using IL as a solvent.

#### 2.2.3. Chitin

Chitin is the second most abundant natural polysaccharide on earth being found in several sources including crab and shrimp shells, arthropod exoskeletons and the molluscan shell of squids. Crystals of chitin are referred to as either alpha or beta chitins with most natural sources being the alpha form. Chains of alpha chitin organize themselves antiparallel using intermolecular hydrogen bonds while beta chitin arranges itself in parallel chains. Beta chitin is held together by weaker molecular forces, making it more susceptible to degradation by enzymes or chemical reactions. Gamma chitin is less studied than the other forms, however research into its physicochemical analysis has shown it to be much more like alpha chitin than beta chitin. One significant difference is that gamma chitin organizes itself into microfibers, whereas other forms organize into nanofibers [103]. Depending on the source, chitin crystals will form fibrils ranging from 2.5 to 25 nm in length. Unlike most other polysaccharides, which are typically neutral or acidic, chitin is highly basic [104]. 

Chitin’s unique optical properties and chelating abilities, combined with its strong mechanical strength and biocompatibility, make it an attractive material for various applications. Common applications include implant devices, wound dressings, drug delivery vehicles and as a component for systems in regenerative medicine. Chitin fibrils can be converted into nanocrystals, nanofibers and nanowhiskers via a top-down method and appropriate work-up. A bottom-up approach can also be used to form gels or self-assembled nano-objects can be regenerated from chitin solutions. Chitin’s mechanical and chemical stability makes it difficult to dissolve in most common solvents, but ILs have frequently been used to dissolve chitin, including for fiber production [105]. Due to the functionality of chitin and chitosan, chitin fibers have many practical applications including in filters [105] and tissue engineering. Of special importance in using ILs to modify chitin’s structure, size and porosity are ILs with short substituents and a cationic ring [106].

#### 2.2.4. Chitosan

Chitosan is a deacetylated derivative of chitin. Structurally, chitosan is a linear chain of glucosamine and *N*-acetyl glucosamine units linked by glycosidic bonds. Chitosan can have varying amounts of glucosamine residues in the polymer chain, which affects the overall properties of the polymer. In order to quantify this, researchers use the degree of deacetylation (DDA), which is the mole fraction of glucosamine residue in the polymer chain [107]. The optimal DDA and molecular weight varies with the intended use of the material, as both of these properties influence the physicochemistry of the molecule including crystallinity, solubility and degradation [104]. Due to the presence of these amines, as well as primary and secondary alcohol groups, chitosan is a highly practical molecule for functionalization. Similarly to chitin, chitosan is also frequently dissolved using IL to convert it into fibers to expand its application potential [19].

#### 2.2.5. Alginate

Another source of polysaccharides is alginate. This is produced by bacteria and seaweed and is used in a large variety of applications due to its unique physicochemistry. Of special note here is its use in advanced pharmaceuticals and biomedical applications within the last couple of decades due to its biocompatibility, nontoxicity and adept uses. Despite both being classified as alginate, bacteria and seaweed alginate have several differences in composition, modifications, molecular mass, viscoelasticity and polydispersity [108]. Molecularly, alginate is a linear, nonbranching polysaccharide consisting of two types of uronic acid residues linked by glycosidic bonds [108]. In nature, alginates usually have a heteropolymeric combination of residues with varying occurrences of β-d-mannuronic acid (M) residues and epimer α-l-guluronic acid (G) residues. Recent research, however, has been able to produce monopolymeric structures using genetically mutated P. aeruginosa bacteria [109,110]. An important note with bacteria-made alginates is that they can be subject to post-translational modifications that cause significant structural changes; bacteria can also naturally acetylate alginates at the O-2 and/or O-3 positions [110]. The resulting composition affects the overall physicochemistry properties of the alginate including viscoelasticity, crosslinking ability [111], strength, stability, mechanical properties [111,112,113,114], solubility, water capacity and molecular mass [109,115,116,117]. These unique qualities lead to a wide range of applications including alginate’s use in nanoparticles, nanotubes, microspheres, microcapsules, sponges, hydrogels, foams, elastomers and fibers [118,119,120,121,122,123,124,125,126,127,128]. Recently, IL has been used to dissolve alginates with other biopolymers to form stable biomaterials [129,130].

### 2.3. Protein and Polysaccharide Based Composite Materials 

In biological systems, most structural materials are composites formed from a dispersed phase, typically biomacromolecules arranged in a hierarchical assemblage. For example, in wood, cellulose is in the dispersed phase, and interacts within a matrix composed of other polysaccharides such as xylan and lignin. In another example, the exoskeleton of arthropods, chitin, another polysaccharide, is in the dispersed phase within a matrix of silk-like proteins. Recent reports characterizing the fabrication of biomaterial composites (biocomposites) using natural materials such as silk, cellulose, bacterial cellulose and chitosan have identified changes in physicochemical and morphological properties as a function of the fabrication method and material composition [131,132,133]. However, the relationship between hierarchical and secondary structures during materials formation is important but sill astonishingly unclear. An important obstacle in the fabrication of biocomposites is an inability to predict the relationship between the complex hierarchical structure and the physicochemical properties of a biomaterial. The ability to manipulate molecules to form hierarchical structures with precise control, size, spacing and shape is a central requirement for achieving the goal of the rapid fabrication of multi-level structures from single structures. In some protein-polysaccharides composites, beta-sheet crystallites provide crosslinks and enhanced mechanical properties. However, their morphology is dependent on preparation conditions and material composition. Currently, the means to control the development of characteristics such as crystallite size and shape are still lacking. Achieving this level of understanding and control will be crucial for progress leading to the ability to fabricate robust multi-level structural biocomposites such as micro-/nanofibers. Understanding molecular self-assembly behaviors and spatiotemporal morphologies in multi-level structural biocomposites will be essential to defining and characterizing the basic phenomena and mechanisms that control the morphology and physicochemical properties and finally the material’s usability.

## 3. Fabrication Methods to Control Fiber Formation 

### 3.1. Solvents Useful for Biopolymer Dissolution 

#### 3.1.1. Ionic Liquids as Solvents

Ionic liquids, traditionally known as room temperature ionic liquids (RTILs), are molten salts that have a melting temperature below 100 °C [134]. Ionic liquids have a wide range of uses in organic synthesis because they can serve as a solvent for many substances, including recalcitrant biopolymers such as cellulose. RTILs have this property because they are made up of polar organic and inorganic components. They have an evaporation temperature greater than 127 °C, and a density greater than that of water. Additionally, they are known as green solvents, meaning that they are able to be regenerated and reused multiple times [135].

Ionic liquids are ideal solvents for the dissolution of polysaccharides and proteins and are commonly used in biopolymer synthesis. Studies have shown that imidazolium derivates are able to blend different proteins and polysaccharides together to create biomaterials, such as biofilms and biofibers. They do this by dissolving both components, denaturing the secondary structure and creating intermolecular interactions without changing the molecular weight or the primary structure. In comparison with traditional organic solvents, Wang et al. demonstrated that ionic liquids successfully dissolve both polysaccharides and proteins without altering the molecular weight [49]. Of particular interest are ionic liquids that have been used to blend together silk and cellulose. Frequently used ILs are 1-allyl-3-methylimidazolium chloride (AMIMCl), 1-butyl-3-methylimidazolium chloride (BMIMCl), 1-Ethyl-3-methylimidazolium chloride (EMIMCl), 1-allyl-3-methylimidazolium acetate (AMIMAc), 1-butyl-3-methylimidazolium acetate (BMIMAc) and 1-Ethyl-3-methylimidazolium acetate (EMIMAc). Table 1 provides a legend of ionic liquid names, abbreviations and chemical structures commonly used in biopolymer studies. 

**Figure 2:**
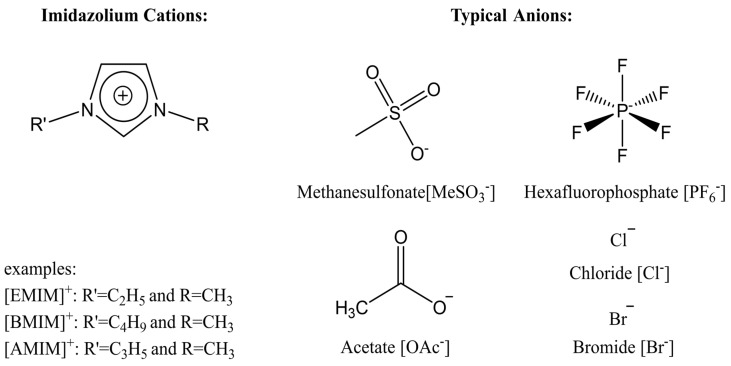
Commonly used ionic liquids for fabricating protein and polysaccharide fiber materials.

Figure 2 illustrates the cations and anions of these ionic liquids. Each ionic liquid contains an imidazolium cation that has a variable hydrocarbon side chain. Anions found in ionic liquids are mostly monatomic excluding organic and inorganic polyatomic ions such as acetate, sulfonate and hexafluorophosphate. Each cation combines with an anion to form an ionic bond [136].

Another advantage of using ionic liquids to dissolve and blend proteins and polysaccharides is that they can be washed from the blend using a polar solvent and regenerated for reuse in subsequent experiments. The most commonly used solvents are distilled/deionized water, ethanol and hydrogen peroxide [136]. The liquids can be separated via simple evaporation/distillation because of the apparent differences in boiling points of the two liquids. This makes the regeneration process relatively inexpensive and sustainable. The variations in the structure of ionic liquids can be used to control the physio-chemical properties of the subsequent created nanoparticles. 

In one study [25], a 10:90 ratio of *B. Mori* silk–cellulose films was investigated as a function of ionic liquid type. Six types of ionic liquids were used in this study, including AMIMCl, BMIMCl, EMIMCl, EMIMAc, 1-Butyl-3-methylimidazolium bromide (BMIMBr) and 1-Butyl-3-methylimidazolium methane sulfonate (BMIMMeSO_3_). Scanning electron microscope (SEM) images provided insight into the surface structures of the blended materials: As a function of ionic liquid type, coagulation carried out in AMIMCl, BMIMCl or EMIMCl produced uniform structures; the EMIMAc film contained porous regions, whereas the BMIMBr and the BMIMMeSO_3_ films both appeared to have a more crystalline morphology. It was also observed that most of the morphology changes occurred as a function of anion type in ionic liquids. 

#### 3.1.2. Other Potential Solvents

Although ionic liquids are quickly becoming the most common solvent for biopolymers, there are several other common solvents that can dissolve recalcitrant organic and biological compounds. In 2005, Kulpinski reported that cellulose nanofibers with diameters of 200–400 nm were produced via electrospinning when *N*-methylmorpholine-*N*-oxide (NMMO) was used as a solvent [137]. NMMO is a heterocyclic amine oxide and morpholine derivative used in organic chemistry as a co-oxidant. The monohydrate is used as a solvent for cellulose in the Lyocell process to produce cellulose fibers. It dissolves cellulose to form a solution called dope, and the cellulose is reprecipitated in a water bath to produce a fiber [138].

Lithium chloride and other alkali solvents have been used to prepare a variety of organic molecules into solution. In particular, lithium chloride (LiCl) and dimethylacetamide (DMA) are known to form an ionic complex that can dissolve the crystalline and amorphous regions of cellulose, chitin and other polysaccharides [139]. At higher concentrations of LiCl, there is more opportunity for polysaccharides to complex through carbonyl groups or other side chain groups. This allows, in turn, more polysaccharide to be dissolved and then precipitated into a biomaterial.

LiCl has also been used in various biomaterial applications of silk. Within tissue engineering, the ability to control a biomaterial’s microarchitecture is vital to a successful scaffold implant. In one study, the microarchitecture of silk sericin was precisely controlled using photolithographic fabrication to guide the adhesion of osteoblasts [140], and the solvent used in this study was a mixture of LiCl and dimethyl sulfoxide (DMSO). In a similar study, photolithography was used to pattern thin films with controllable degradation made from silk proteins dissolved in LiCl/DMSO [141]. The same patterning techniques have also been used to create conductive silk biocomposites for degradable bioelectronic sensors [142].

Methanoic acid, colloquially known as formic acid, is a common solvent used in the synthesis of biopolymers. The hydrogen ions in formic acid work to interrupt the hydrogen bonds present in the backbone of many organic molecules, disrupting their native structure and allowing the polymer to dissolve into solution. In one study [143], formic acid was shown to dissolve both corn zein and silk fibroin, which could then be spun into nanofibers. Fourier-transform infrared (FTIR) spectroscopy results show how formic acid was able to modify the protein structure of zein from its native, random coil structure into a more ordered alpha-helical structure in its fiber and film forms.

In several studies [17,144], formic acid was mixed with calcium chloride to break down natural silk by disrupting the native structure and breaking down carbon-carbon and carbon-nitrogen chains. Prolonged exposure to formic acid lowered the molecular weight of the silk and affected the thermal stability of the final biopolymer, including its glass transition temperature and region. Similar results could be expected with other natural biopolymers.

Aqueous tetrabutylammonium hydroxide (TBAOH) has recently received attention for being an efficient alternative to common organic solvents. In the literature, it is commonly used to drive cellulose into solutions with concentrations as high as 10 wt% [145,146]. At low concentrations (2 wt%), TBAOH is able to dissolve cellulose in minutes at room temperature. Interestingly, this solvent gives some control over the cellulose–cellulose interactions in solution, as cellulose is repulsive in dilute solutions, but attracts into aggregates at concentrations above 0.04 g cm^−3^. In this scenario, cellulose I is soluble, while cellulose II precipitates into aggregates. TBAOH is also a plausible solvent for other biopolymers including silk fibroin in concentrations up to 60 mg mL^−1^ without adding heat [147].

### 3.2. Fiber Spinning Methods 

#### 3.2.1. Electrospinning 

Electrospinning is a common method to create micro/nanoscale fibers from biopolymers, which are often difficult to produce in a simple, cost-effective manner [148]. Electrospinning was first patented by Anton Formhals in 1930 [149]. Electrospinning gives unique properties to nanofibers such as large surface area, lower structural defect and enhanced mechanical properties [150]. Most electrospinning devices are made up of three components: a syringe pump that holds the polymer solution, a high voltage electric source that creates an electrostatic field and draws the solution into a fibrous jet stream and a grounded target apparatus that collects the nanofibers [136]. The syringe can be attached to a motor that can control and stabilize the rate that the polymer solution is fed to the system. The target apparatus can be modified in several ways to allow for post-production solvent removal or physiological modifications to the fibers.

Prior to starting the electrospinning process, the biopolymers are dissolved into a solvent and placed into a syringe. The pump then forces the polymer solution towards the tip of the syringe at a constant rate, where it remains as a drop due to surface tension. The high-voltage electrostatic field induces charges in the solution and draws the solution into a Taylor cone. As the charged solution continues to interact with the electric field, the electrostatic forces overcome the surface tension forces and a fiber stream elongates from the stable Taylor cone and travels toward the target apparatus [136]. The stream begins to bend and form large spiral loops as the length increases due to increased instability. The diameter of the loop is inversely proportional to the diameter of the jet but directly proportional to the length. Under ideal conditions, the solvent will evaporate as the stream travels to the target site, leaving behind polymer fibers in the range of 10 nm to 10 μm due to the high surface area to volume ratio. The electrospinning setup can be adjusted to modify the structure of the nanofibers.

It is important to note that the appearance of the fibers from the Taylor cone is impacted by several different factors. When the solution initially leaves the cone, it follows a straight path, but it quickly takes on one of three modes based on instabilities in the stream [21]. The instabilities, as described by Shin et al. in 2001, are either axis-symmetric, where fluctuations in the fiber stream occur in the central axis, or non-axis-symmetric. The first of the three modes is Rayleigh instability. This mode is produced when the solution has a high surface tension, and it is suppressed in a high electric field. When the stream exhibits Rayleigh instability, droplets are formed instead of a continuous stream. This is known as electrospraying. The second axis-symmetric mode occurs when the polymer solution is highly viscous, leading to the formation of beads in the fiber stream. Lastly, the non-axis-symmetric mode allows for the stream to bend into the ideal spiral loop of nanofibers. This occurs when the solution contains a high surface charge density and has a high fluid flow rate. When the stream exhibits non-axis-symmetry, the stream will be thinned and smooth nanofibers will be produced [151].

**Figure 3:**
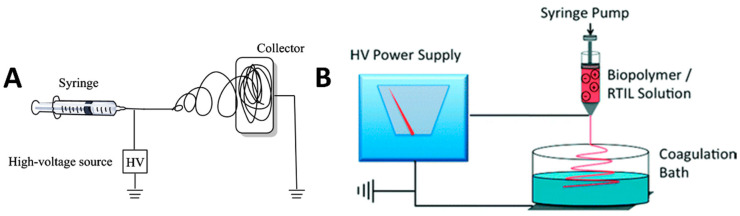
Examples of electrospinning setups utilized with ionic liquid solvents. (**A**) Basic horizontal electrospinning setup, with a polymer solution-fed syringe, a high-voltage source and a grounded collector plate. (**B**) Wet spinning, where fibers are spun into a coagulation bath instead of a grounded collection plate. (**A** is reproduced with permission from Ref. [154]; Copyright 2014 Elsevier. **B** is reproduced with permission from Ref. [21]; Copyright 2010 The Royal Society of Chemistry).

The electrospinning setup can play a large role in many of the above-mentioned factors. Due to this, many different variations have evolved from the original horizontal electrospinning setup. Figure 3 illustrates the basic horizontal electrospinning setup (Figure 3A) and a wet spinning setup for IL-based samples (Figure 3B), where a polymer is electrospun directly into a coagulation agent [21]. Other setups include a vertical format of the standard setup, which helps to counter uncooperative electrostatic forces and changes the morphology of the fibers produced [151]. Vertical setups have been further modified by the same author, by utilizing a rotating drum as a collection plate and a rotating disc to further modify the morphology of the fibers produced. Several different modifications of the horizontal electrospinning setup also exist in recent literature: A side-by-side syringe setup is one of these, where two syringes filled with polymer solution are used simultaneously in order to combine the benefits of two different polymers that may not be compatible with the same solvent [152]. Modifications to the collection plate are also used, such as using parallel plates as a collection target, in order to better control the electrostatic forces that affect the polymer trajectory [131]. Another of these modifications builds upon the rotating drum concept by adding a translation element to the drum to further modify the fiber morphology [153]. Modifications to the syringe have also been used in recent works, where a process called melt-spinning is used to modify the polymer solution viscosity prior to electrostatic forces taking control [132].

**Figure 4:**
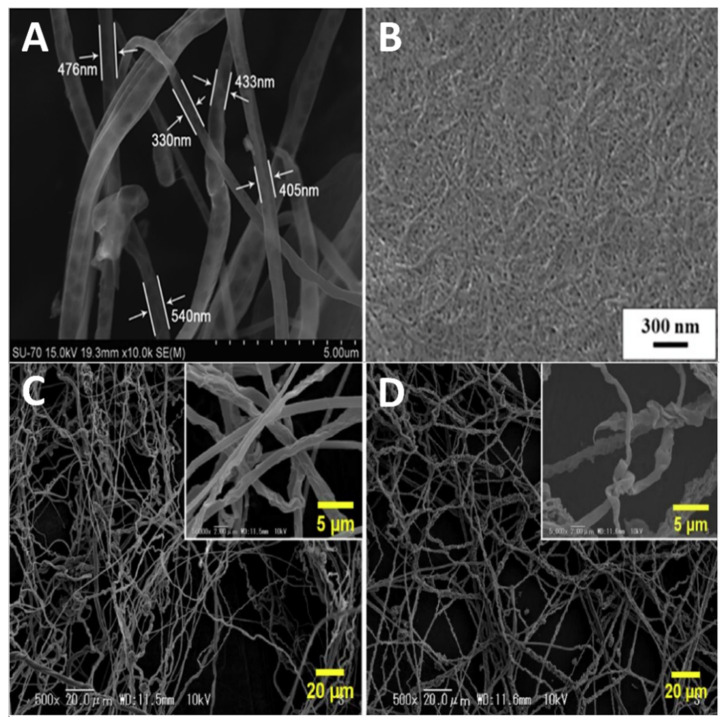
SEM images of electrospun nanofibers made from (**A**) cellulose from [C_2_MIM][CH_3_CO_2_], (**B**) chitin from [C_2_MIM][OAc] and (**C**) 4.8 wt% and (**D**) 16.7 wt% cellulose made from [BMIMCl]. (**A** is used with permission from Ref. [98]; Copyright 2011 The Royal Society of Chemistry. **B** is used with permission from Ref. [155]; Copyright 2016 European Chemical Societies Publishing. **C**, **D** are used with permission from Ref. [99]; Copyright 2018 Elsevier).

In addition to solution viscosity, flow rate, surface charge density and electric field strength, the spinnability of a biopolymer solution can be controlled by several other parameters related to the spinning technique and the solution itself. Even the choice of ionic liquid itself can affect the characteristics of the fibers formed. Critical spinning parameters include the distance between the syringe and grounded target, the feed rate and the electric field strength. The most important solution parameters are viscosity, surface tension, polymer concentration and molecular weight, mass distribution and chemical structure. The last four parameters directly affect the chain entanglement density and spinnability of the polymers [21]. SEM images of a few example nanofibers are shown in Figure 4.

In the literature, many studies are focused on controlling the physicochemical properties of nanofibers. One of the most important properties is the diameter of the fiber. Ambient temperature and humidity are two parameters that can affect this property. Pelipenko et al. observed that the diameter of electrospun fibers decreased as the ambient humidity increased [156]. In their experiments, fibers were created from polyvinyl alcohol (PVA) and polyethylene oxide (PEO) polymer solutions as well as PVA/hyaluronic acid (HA) and PEO/chitosan (CS) blended polymer solutions. They observed that the diameter of all the nanofibers decreased as the ambient humidity increased. It was also demonstrated that the morphology of the fibers changed as a function relative to humidity [156,157]. As the ambient humidity increased, beads and pores began to develop in the structure of the fibers. In some cases, the increased humidity prevented nanofibers from forming all together and only allowed for electrospraying [158]. This occurs because polymer solutions tend to retain more water when the humidity increases. Increased humidity levels also prevent solvent evaporation as the polymer stream is traveling towards the collector [156].

The ambient temperature also affects the diameter of the nanofibers, as reported by De Vrieze et al. [156]. In their experiments, nanofibers were electrospun from polyvinylpyrrolidone (PVP) and cellulose acetate (CA) polymer solutions. The PVP was dissolved in ethanol, while CA was dissolved in acetone and dimethylacetamide (DMAC). They observed that the average diameter of the nanofibers spun at 283 and 303 K was lower than those spun at 293 K, when the relative humidity and the distance between the tip of the syringe and the collector were kept constant. This phenomenon can be explained by two opposing effects: At lower temperatures, the solvent dissolves at a much slower rate. Subsequently, the polymer will need more time to solidify which means the stream will continue to elongate. On the contrary, at higher temperatures, the solution is less viscous which makes it more susceptible to higher stretching and thinning. Bae et al. and Yang et al. reported similar thermal and barometric results in their studies [158,159].

#### 3.2.2. Wet Spinning/Dry-Jet Wet Spinning

Wet spinning is a cost-effective fiber fabrication technique that allows for the elongation of a polymer solution in a coagulation bath. In this method, the polymer solution is pumped through a syringe directly into a bath that removes the solvent and allows the polymer to precipitate into a fiber [159]. The fiber can then be elongated by applying tension to the stream and drawing it to the desired length [160]. The setup can be modified to include multiple wash baths and drawing systems to improve the molecular alignment and orientation [161].

An alternative setup of this technique is call dry-jet wet spinning. In this method, the jet stream is first elongated in air before going into the coagulation bath. This allows for some of the solvent to evaporate, resulting in greater molecular alignment. [162].

In contrast to electrospinning, these two techniques do not use an electric field to elongate the fiber stream. This allows for the fibers produced by these methods to have enhanced molecular alignment, but the diameters will be on the micron scale compared with the nanoscale with electrospun fibers [3,160].

#### 3.2.3. Phase Separation

Phase separation involves dissolving a polymer system with a solvent and creating a gel from the solution by decreasing the temperature. Once the solution is sufficiently cooled and solidified, the resulting gel can then be soaked in distilled water, which will extract the solvent. Nanofibers will form once the gel is blotted with filter paper and freeze-dried. The structure of the fibers can be controlled by adjusting the concentration of the polymer and the temperature at which the gel is formed [163].

## 4. Impact of Coagulation and Ions on Material Structure

Once the fibers are produced, several post-treatment steps, including chemical coagulation, can be applied to further enhance or study the morphology and induce ion conductivity of the materials. 

### 4.1. Chemical Coagulation

Post-production chemical treatments are applied to biopolymer nanofibers to help further extract any remaining ionic liquid from the samples. Removal of the solvent helps to further stabilize and coagulate the nanofiber. Some of the common chemicals used as a wash are water, methanol and H_2_O_2_.

#### 4.1.1. Water

**Figure 5:**
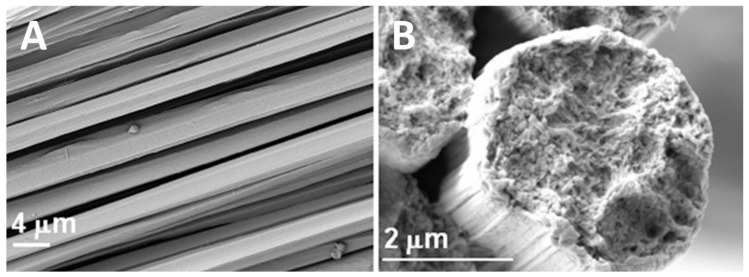
SEM images of regenerated cellulose fibers using ionic liquids and a water-based coagulation bath: (**A**) surface image; (**B**) cross-section image [100]. (Reproduced with permission from Ref. [100]; Copyright 2018 Wiley).

Water is an ideal solvent to wash ionic liquids from the surface of the nanofibers. The high polarity of the molecule easily extracts the ionic liquid solvent. When the solvent is removed, the nanofiber will further coagulate and stabilize. This is because the biopolymers are separated due to their electrostatic interactions with the anions in the ionic liquid. The removal of the solvent allows for the biopolymers to interact with each other using hydrogen bonding and causes them to aggregate into a fiber [25]. In addition to ionic liquid extraction, water also helps to modify the diameter and structure of the nanofibers. In 2017, Grimmelsmann et al. reported that the morphology and diameter of chitosan/poly(ethylene oxide) nanofibers was altered after being washed for 30 s in deionized water [164]. Even though water is ideal, one disadvantage is that it can replace ionic liquid and form hydrogen bonds with the biomolecules. This will lead to competition between adjacent biopolymers and water not able to extract any ionic liquid from within the nanofiber. Any solvent trapped within the fiber will be stabilized by the electrostatic attraction to the polymer and protected by the hydrophobic interactions with water. Figure 5 shows cellulose fibers prepared from cellulose dissolved in ionic liquid and wet-spun directly into a water coagulation bath to form highly crystalline fibers. 

In another study on *B. Mori* silk-cellulose composites, it showed that water coagulation can change the structure of cellulose microcrystalline, and the β-sheet content of silk can be manipulated by disrupting inter- and intra-molecular hydrogen bonds during the coagulation. The result suggested an intermediate semi-crystalline or amorphous structure in the composites, which was confirmed by X-ray scattering [165]. Molecularly, this demonstrated that the cellulose microfibril diameter decreased as the silk content increased within the composite of cellulose and silk. As cellulose content increased, β-sheets size also increased; even a small percentage of silk (10%) into cellulose caused disruption of the cellulose structure and the assembly into cellulose I structures.

#### 4.1.2. Methanol and Other Organic Solvents

Methanol is a simple alcohol consisting of a methyl group linked to a hydroxyl group, while ethanol, also known as ethyl alcohol, consists of an ethyl group linked to a hydroxyl group. Both alcohols are used to induce the formation of biopolymer fibers after ionic liquid dissolution. The highly polar agents act to remove the ionic liquid from the solution due to its hydroxyl (OH) group and high electronegativity of oxygen, allowing hydrogen bonding to take place with other molecules. The attraction to non-polar molecules makes it an ideal solution to interact with the polar and non-polar regions of biopolymers and ionic liquids, fusing the materials. In addition, the protein conformational transition change from random coil to β-sheet form is known to be induced by the methanol treatment.

**Figure 6:**
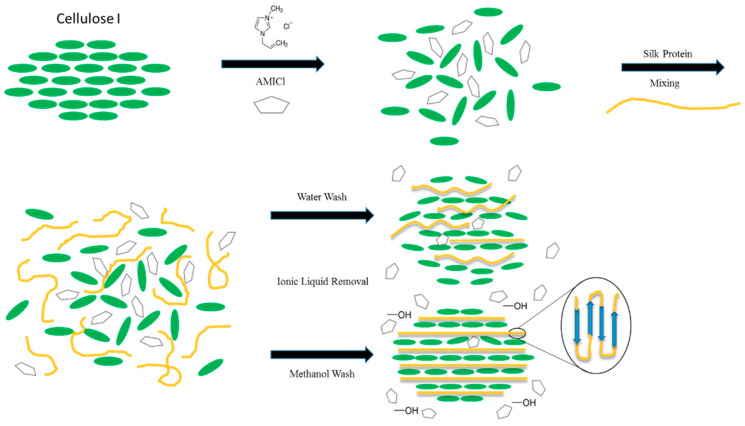
Water and methanol coagulation agents have different effects on the self-assembly of Thai silk-cellulose polymer composites. (Reproduced with permission from Ref. [102]; Copyright 2017 Elsevier).

The structural differences between using water or using methanol as a coagulation agent was observed in the *Thai* silk-cellulose polymer (Figure 6) [102]. Thermal analysis showed that the thermal and physical properties could be finely tuned by manipulating hydrophobic–hydrophobic or electrostatic interactions between the silk and cellulose. Upon dissolution in ionic liquid, cellulose assumes a disordered structure with expanded fibrils that easily interact with silk molecules. After washing out the ionic liquid with a coagulation agent however, the unorganized cellulose reverts to a crystalline cellulose I structure with silk molecules inserted within, as confirmed by FTIR analysis. This process proceeds via immiscible phase separation. When methanol is used as a solvent, the silk components crystallize, resulting in a large number of β-sheets within the composite. Due to this, composites with any amount of silk will generally be mechanically superior to pure cellulose samples when treated with methanol instead of water. Further insight into these coagulation mechanisms opens up the possibility of fine-tuning biopolymer composites through the use of different coagulation agents. 

Most water-insoluble polysaccharide solutions can be coagulated in both the abovementioned solvents (water or organic solvents). Protein solutions, on the other hand, are usually coagulated in organic solvents such as alcohols since most proteins are soluble in water. In one study, ethanol was used to regenerate cellulose/silk fibroin from *N*,*N*-dimethylacetamide/LiCl (DMAC/LiCl). Even though there was no visible phase separation, micro-voids and a low degree of crystallinity in the blend structures were reported [48]. Thus, it can be understood that these differences in blended structures can be due to the different requirements of coagulation for silk fibroin and cellulose.

#### 4.1.3. H_2_O_2_ Coagulation

Hydrogen peroxide (H_2_O_2_) is another chemical agent that induces the coagulation of biopolymers. Hydrogen peroxide is the simplest peroxide consisting of an oxygen–oxygen single bond. It is highly unstable and slowly decomposes in the presence of light. The mixture of hydrogen peroxide and water is useful to wash out ionic liquids due to its interaction with the anions in the solution and ability to form hydrogen bonds. Depending on its ratio of water to H_2_O_2_, its distinct use on biopolymers such as cellulose induces a highly crystalline and brittle material.

In a study by Love et al. [166], 1-ethyl-3-methylimidazolium acetate (EMIMAc) ionic liquid was used to dissolve silk and cellulose into a composite biomaterial. The materials were then regenerated with either water or varying percentages of hydrogen peroxide (1–25%). EMIMAc was able to completely dissociate both silk and cellulose into solution and was casted into thin films. Following casting, the ionic liquid was easily washed out by the coagulation agent used, either water or hydrogen peroxide. The impact of ionic liquid as a solvent on the material’s morphological, thermal, mechanical and electrical properties was studied. Thermal and morphological analysis both showed that a higher percentage of hydrogen peroxide promoted hydrogen bonding between hydroxide groups on cellulose, resulting in increased crystallinity and crystal size in the final biomaterial.

### 4.2. Effect of Ionic Liquid Ions on Fiber Structure and Ion Conductivity

**Figure 7:**
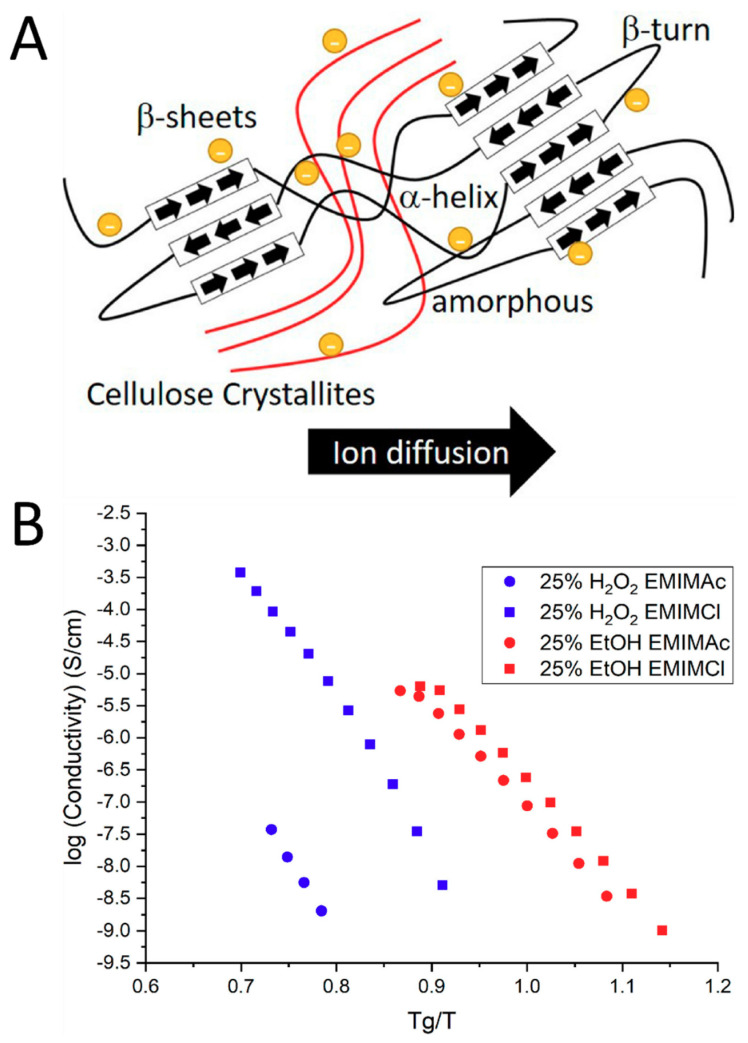
(**A**) Schematic representation of how ion diffusion through a solid electrolyte is dependent on the molecular structure, including the content of various protein structures. (**B**) Choice of coagulation agent affects the morphology of a polymer, which in turn affects its ability to conduct ions. Results are normalized to the glass transition temperature of each polymer. (Reproduced with permission from Ref. [25]; Copyright 2019 Society of Chemical Industry).

The inter- and intramolecular interactions between the anions of the ionic liquid and the hydroxide groups in natural polymers can cause changes in the distribution of secondary structures in the polymer. For example, using EMIMAc as a solvent will give a different protein sub-structure than using EMIMCl due to different effects from the acetate anion or the chloride anion [25]. In this study, using EMIMCl as a solvent resulted in more beta sheets in the final biomaterial compared with using EMIMAc. Another study showed that bulkier anions, such as BMIMBr and BMIM-MeSO_3_ can increase molecular interactions because of their larger size. These molecular interactions include electrostatic, hydrogen bonding and hydrophobic–hydrophobic interactions between biopolymers in solution [165]. Changing these structures leads to changes in the polymer’s overall morphological, thermal and mechanical properties, indicating that ionic liquid choice can be used to tailor biopolymers towards specific applications. Morphological changes also lead to ionic conductivity modifications, which differed with the coagulation baths. These changes can influence the ionic conductivity of the polymer in a way illustrated in Figure 7. Previous studies [167] have shown that beta sheet content of proteins is correlated with ionic conductivity. Connecting these two ideas shows how ionic liquid choices can be important in the production of batteries and bioelectronics.

In a similar study by Mahmood and colleagues [24], in a process termed “natural fiber welding”, the ionic mobilizes fibrous polymers at their surface by disrupting hydrogen bonds. These mobile materials are then intertwined with materials from neighboring fibers and form a uniform layer after removal of the ionic liquid solvent. Trulove et al. utilized this process with *Bombyx mori* silk and hemp thread. Their X-ray diffraction (XRD) and infrared (FTIR) results showed that significant amounts of the native structure of the polymer were retained [168]. They go on to show that the most impactful factor of this process was the amount and location of the modification to the polymer. Some researchers have even used this technique to functionalize fibers at the surface using chemical modifications to the molecule’s sidechains [169,170].

Mathematically, the morphology and structure of a polymer relate to its ability to dissociate ions through a combination of the Arrhenius equation and the Vogel–Fulcher–Tammann Equation (1):

(1) $$\sigma =qp_{\infty }\mu_{\infty }exp\left(-\frac{E_{a}}{\bar{R}T}\right)exp\left[-\frac{B}{\bar{R}\left(T-T_{0}\right)}\right]$$

In Equation (1), *q* is the charge of the ions, *p_∞_* is the total ion density, *µ_∞_* is ion mobility, *E_a_* is the activation energy, *T* is the temperature, *R* is the universal gas constant, *B* is an energy barrier constant and *T*_0_ is the Vogel temperature [22,171,172]. The energy barrier constant *B* is a sum of the energy barrier for polymer segmental motion and the energy barrier of ion hopping [173]. The latter is worth discussing in the context of polymer morphology as it scales with the square of the hopping distance of the ion. This is true in both intramolecular and intermolecular motions, meaning the side-chain length of polymers, a function of protein sub-structures and polysaccharide side-chain groups, plays a significant role in the ionic conductivity of a biopolymer. In the context of IL, the energy barrier of ion hopping also scales with the size of the ion, which can be selected for when choosing an IL for a particular biomaterial. Another mechanism that affects ion diffusion and dissociation is the segmental movement of sidechains in the polymer [22,174,175]. All of these morphological processes can be attributed to the energy barrier constant in Equation (1), thus showing how the morphology of a polymer can have a massive effect on its ion conductivity. This concept is illustrated in Figure 7 through different ILs ratios and different coagulation agents. 

## 5. Novel Applications of Biopolymer-Based Fibers from Ionic Liquids

### 5.1. Tissue Regeneration

Ionic liquids have recently found their way into nanofibers intended for biomedical applications. In 2014, AMIMCl was used as a solvent in the fabrication of a cellulose-based tissue engineering scaffold with a tunable microarchitecture [176]. These scaffolds were tested by culturing fibroblast cells on them for 15 days to compare the biocompatibility of the samples to a control 2D cell culture dish. A live/dead assay and DAPI staining were also performed to evaluate the biocompatibility in vitro. Over nine days, there was continuously a higher number of cells attached to the scaffold, and the MTT assay confirmed that the cells were metabolically active. DAPI staining was also used to show cell adherence to the scaffold.

**Figure 8:**
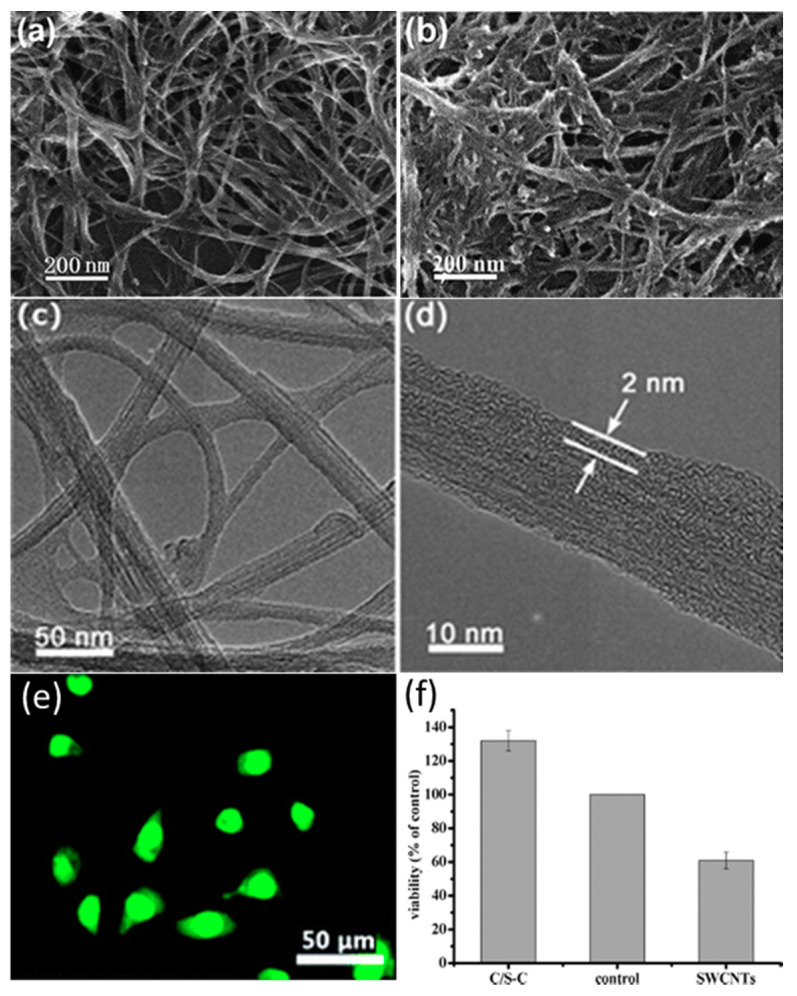
(**a**,**b**) Field emission SEM and (**c**,**d**) high-resolution TEM micrographs of cellulose/single-walled carbon nanotube (SWCNT) complexes (C/S-Cs) generated through an ionic liquid (BMIMBr); (**e**) fluorescent microscopy of HeLa cells after 24 h of growth and acridine orange (AO) and ethidium bromide (EB) staining; (**f**) WST-1 assay shows a significant increase in HeLa cell viability on C/S-C. (Reproduced with permission from Ref. [177]; Copyright 2009 The Royal Society of Chemistry).

In a similar vein of work, cellulose was able to be combined with single-walled carbon nanotubes (SWCNTs) in order to form a biomaterial with high biocompatibility [177]. This was accomplished in part from cellulose’s ability to easily dissolve in 1-butyl-3-methylimidazolium bromide (BMIMBr). The cellulose/SWCNT complexes (C/S-Cs) were characterized through field emission SEM (Figure 8a,b), high-resolution TEM (Figure 8c,d) and FTIR. Their superior biocompatibility was confirmed by a WST-1 assay using HeLa cells as well as acridine orange (AO) and ethidium bromide (EB) double staining. Figure 8e shows the fluorescent microscopy of healthy HeLa cells growing on C/S-Cs complexes. The lack of red EB dye indicates that no cells are in a necrotic state. Figure 8f shows the results of the WST-1 assay, which shows a clear increase in cell viability in C/S-Cs complexes compared with both the glass microscope slides and purified SWCNTs without cellulose wrapping.

### 5.2. Drug Delivery

**Figure 9:**
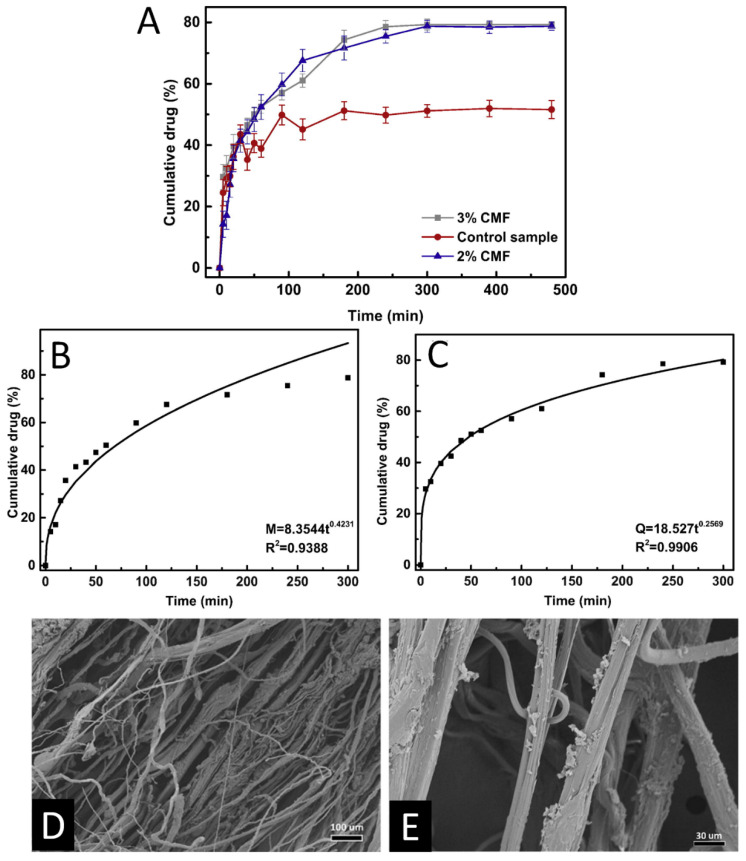
Drug release profiles for (**A**) IBU@2% cellulose micro-nanofibers (CMFs) and IBU@3% CMF matrices compared with a control tea bag and the curve fits based on the Peppas equation for the (**B**) 2% and (**C**) 3% samples; (**D**,**E**) SEM images of cellulose micro-nanofibers (CMFs) IBU@3% matrices. (Reproduced with permission from Ref. [178]; Copyright 2017 Elsevier).

An area of expertise for nanofibers formed from ionic liquids is topical drug delivery. To cite a specific example, Liu and colleagues electrospun fibrous membranes from regenerated cellulose dissolved in 1-butyl-3-methylimidazolium chloride (BMIMCl) (Figure 9) [178]. The resulting cellulose micro-nanofiber (CMF) matrix was soaked in a solution of ibuprofen in ethanol in order to load it with the drug to test its capabilities as a drug carrier. Material characterization was performed through SEM, FTIR, TGA, tribological testing wettability and contact angle testing, 24-h cell viability assay and a drug release assay. Nanofiber diameters ranged from under 1 to around 20 µm with limited morphological differences between 2 and 3% wt/vol of ibuprofen. At 2%, however, there was notable fusion that occurred between the ionic liquid surfaces and water surfaces due to the ionic liquid not washing off fast enough during the electrospinning process. This fusion was mostly absent in the 3% samples. At 3%, the integration of ibuprofen shifted the thermal degradation of the fiber matrixes slightly higher due to hydrogen bonding. Tribological testing was performed with the intent to minimize roughness which would irritate the skin if the fibers were used as a bandage. Overall, the research determined through coefficients of friction that the surface roughness was lower than that of other electrospun fibers and drug loading did not significantly increase the coefficient. SEM images of these fibers are shown in Figure 9D,E, with different scale bars. A 24-h cell-viability test classified the materials as qualified biomaterials under the United States Pharmacopeia, with a higher viability than a control cellulose material. After this qualification, a drug release study was done under physiological conditions of 37 °C and 5.5 pH to mimic the conditions on human skin. In order to model the release of ibuprofen, the drug release was plotted and fitted to a curve based on the Peppas equation. The results for both of these studies are plotted in Figure 9A–C. Release exponents of 0.42 and 0.25 for the 2% and 3% IBU samples, respectively, indicate that both drug releases were controlled by Fickian diffusion through the CFM matrices. The fiber samples exhibited a biphasic release, with a quick initial release for 100 min followed by a slower release to a cumulative 80% drug release for the last 200 min. The researchers noted that this time is ideal for other drug carriers loaded by simple immersing, and that they have the potential to be reloaded and reused.

### 5.3. Water Purification

**Figure 10:**
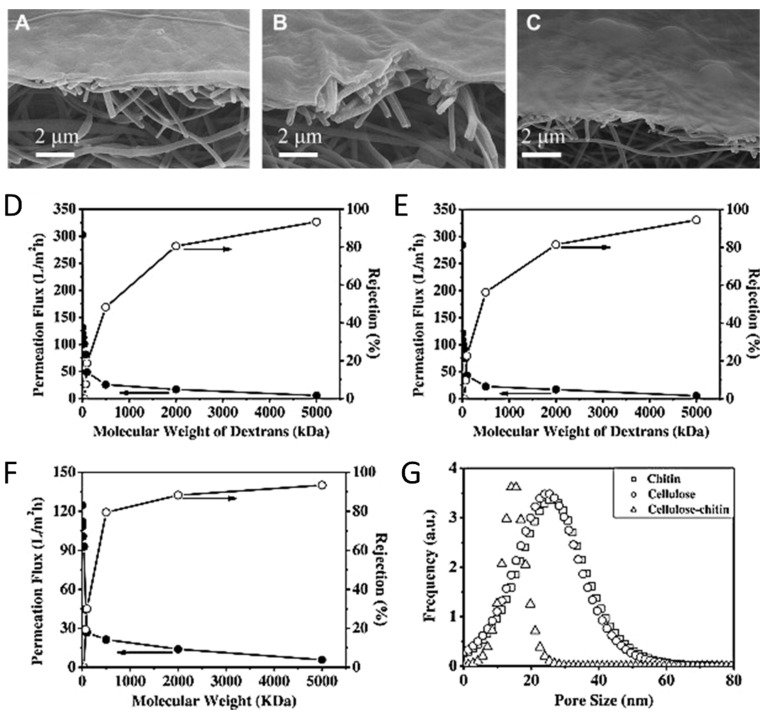
Cross-section SEM images of (**A**) cellulose, (**B**) chitin and (**C**) cellulose–chitin blend barrier layers prepared by ionic liquid regeneration in 1-ethyl-3-methylimidazolium acetate. Graphs (**D**–**F**) show the permeation flux and rejection ratios of (**D**) cellulose, (**E**) chitin and (**F**) chitin-cellulose composite membranes, while (**G**) compares the distribution of pore sizes in the membranes. (Reproduced with permission from Ref. [179]; Copyright 2011 Elsevier).

Polysaccharides, including cellulose, have seen increasing use as a material in wastewater filters recently. However, since cellulose can be digested by microorganisms, it can be combined with chitin, which is more bioinert, to form the barrier layer of a nanofibrous scaffold for filtration. SEM images of this barrier layer are shown in Figure 10 for cellulose, chitin and chitin-cellulose composite barrier layers. High-flux thin-film nanofibrous composite (TFNC) membranes were created using chitin, cellulose and chitin-cellulose composites regenerated from 1-ethyl-3-methylimidazolium acetate (EAc) [179]. The dissolution was then added to ethanol to regenerate the polysaccharide structure. Normally, microcrystalline cellulose is too crystalline to be processed into membranes [180], but the analysis done in this study shows that cellulose and chitin can be used as a barrier layer for membranes since the layer was less than 500 nm thick. The membranes fabricated were able to take advantage of the properties of cellulose and chitin to produce membranes with a high permeation flux and high rejection ratio compared with commercially available membranes. Figure 10D–F also compares cellulose, chitin and chitin-cellulose composite TFNC membranes to show a high permeation flux and a comparable rejection ratio. Figure 10G compares the distribution of pore sizes in the three membranes. The rejection ratio R% was calculated by Equation (2):

(2) $$R\%=\frac{C_{f}-C_{p}}{C_{f}}*100\%$$

where *C_f_* is the concentration of the feed solution and *C_p_* is the concentration of the permeation solution.

In addition to the successful membrane product, the ionic liquid used in this study was able to be recovered and reused via distillation at atmospheric pressure. ^1^H nuclear magnetic resonance (NMR) spectroscopy confirmed the purity of the recycled ionic liquid within 2% impurity, which had no effect on its ability to re-dissolve chitin.

### 5.4. Recycling of Ionic Liquid

**Figure 11:**
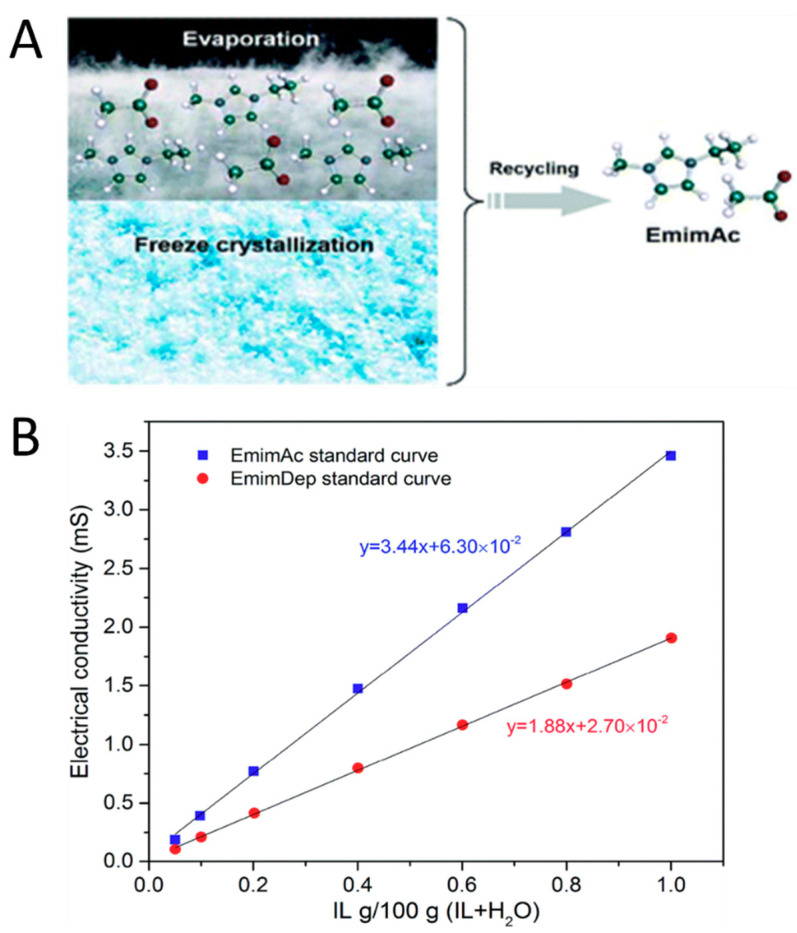
(**A**) A combination of evaporation and freeze crystallization is used to separate and recover ionic liquid mixed with water in this cellulose nanofiber fabrication method. (**B**) Standard conductivity curves for EMIMAc and EMIMDep used to determine the concentration of ionic liquid in the regenerated solvents. (Reproduced with permission from Ref. [101]; Copyright The Royal Society of Chemistry).

One of the most valuable aspects of using ionic liquids as a solvent relates to its inherent green chemistry. After use, ionic liquids can easily be recovered and reused by adding non-solvents such as water or acetone and then evaporating the non-solvent to recover the ionic liquid that was used, such as in Figure 11 [181,182]. Ongoing studies are being done to select suitable non-solvents that maximize the yield of recovered ionic liquid and reduce the overall cost of processing biomass into useful forms. Some methods of recovering ionic liquid currently being studied include column chromatography based on solid adsorption properties [183] with organic solvents and a combination of freeze crystallization and evaporation [101] of water in a mixture of ionic liquid and water.

In one particular example, evaporation and freeze drying were utilized to recover EMIMAc and EMIMDep during the process of cellulose nanofiber formation. A graphical abstract is shown in Figure 11A. Enthalpies involved in each step of the recovery process were used to calculate the energy consumption required to recover the ionic liquid used per kilogram of cellulose dissolved. These calculations also relied on the assumptions that freeze drying had an efficiency of 60%, while vacuum drying and the evaporation process were 100% efficient. Their calculations show that using freeze drying prior to the evaporation of ionic liquid, compared with simply evaporating ionic liquid, is a more energy-efficient process for the recycling of ionic liquid. The recycling process was highly effective, where the EMIMAc and EMIMDep regenerated solvents contained 94.2% and 94.8%, of the original amount of solvent, respectively. These values were determined by the electrical conductivity of the regenerated solvents using a standard calibration curve for each ionic liquid, shown in Figure 11B.

### 5.5. Electromechanical Actuators

An important component of electroactive polymers (EAP) are electromechanical actuators. Many novel EAPs are fabricated using ionic liquids as a dopant within EAP materials. Ionic liquids with cations of large van der Waals volumes (e.g., [C_2_mim][Tf], [TES][NTf_2_], [BMP][NTf_2_] and [C_2_mim][Cl]) have been used as dopants to enhance cationic strain [184], while others like [C_2_mim][PF_6_] and [C_6_mim][PF_6_] are used in actuators to generate large strains of up to 4% [185]. By combining EAPs with ionic liquids, biocompatible electromechanical actuators have been fabricated with effective bending actuation and tunable mechanical properties [184]. Before the use of ionic liquids, EAP would require the use of an electrolyte solution to function properly. The recent use of ionic liquids in this field, combined with ionic liquids’ potential as solvents in fiber formation, shows large potential for bioactive polymers as implants or in medical devices.

## 6. Conclusions

Fiber materials created from natural macromolecules hold great promise in several fields including medicine, green chemistry and bioelectronics. The molecular structure of these fibrous materials can be fine-tuned for specific applications using specific solvents and preparation methods. Recently, the use of ionic liquid as a solvent or in the processing of materials has shown great promise in optimizing and functionalizing biomaterials. Understanding the mechanisms behind fiber formation and biopolymer interactions in ionic liquid will help guide fiber materials into more organized, multi-level 1D fiber materials or fiber matrices for many uses. Using the wide range of ionic liquids and fiber preparation and processing techniques, fiber materials from natural sources have become more robust than ever while maintaining or improving their green footprints.

## Figures and Tables

**Table 1:** Ionic liquid names, abbreviations and chemical structures commonly used in biopolymer studies.

Ionic Liquid	Abbreviation(s)	Chemical Structure
1-Allyl-3-methylimidazolium chloride	AMIMCl	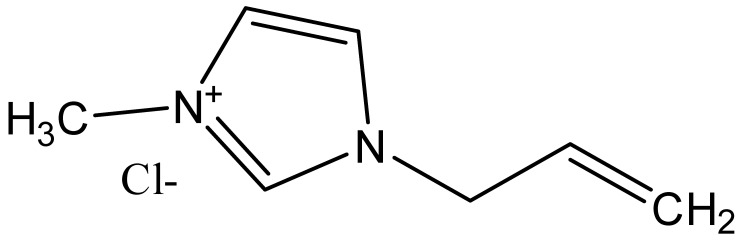
1-Ethyl-3-methylimidazolium chloride	[C_2_MIM][Cl] or EMIMCl	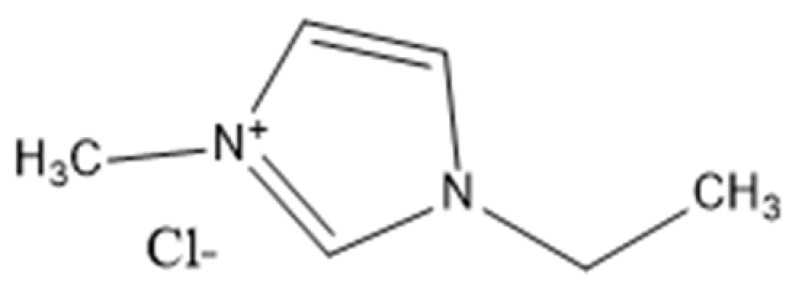
1-Butyl-3-methylimidazolium chloride	[C_4_C_1_IM]Cl or BMIMCl	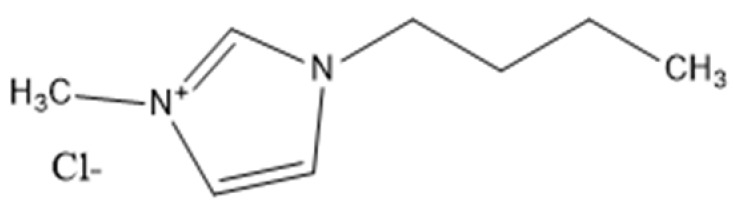
1-Allyl-3-methylimidazolium acetate	AMIMAc	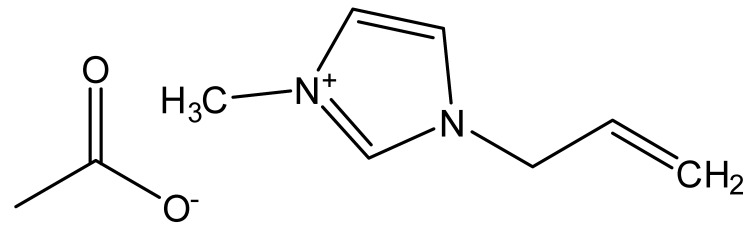
1-Ethyl-3-methylimidazolium acetate	[C_2_MIM][CH_3_CO_2_] or [C_2_MIM][OAc] or EMIMAc	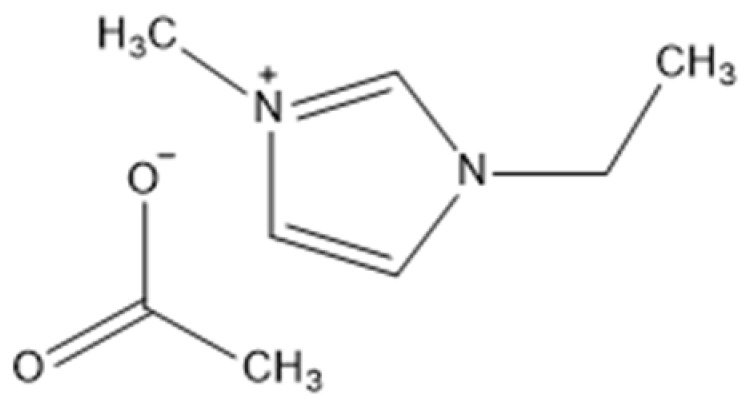
1-Butyl-3-methylimidazolium acetate	BMIMAc	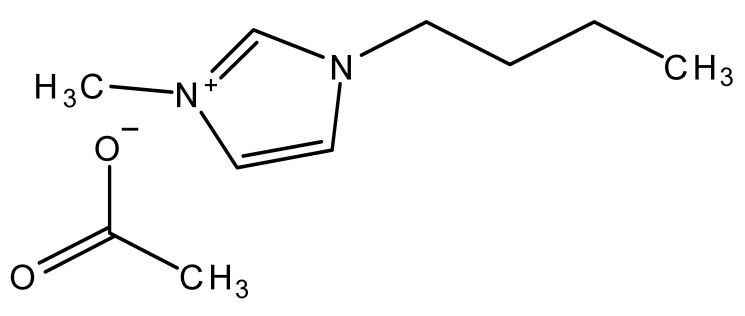
1-Butyl-3-methylimidazolium dicyanamide	[BMIM][DCA]	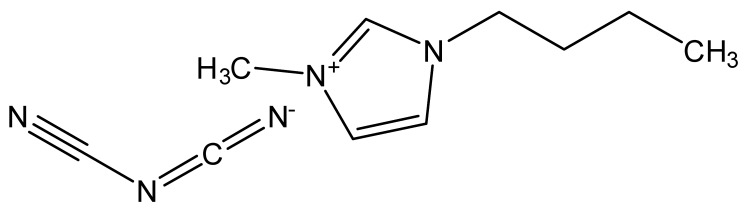
1-Butyl-3-methylimidazolium bromide	BMIMBr	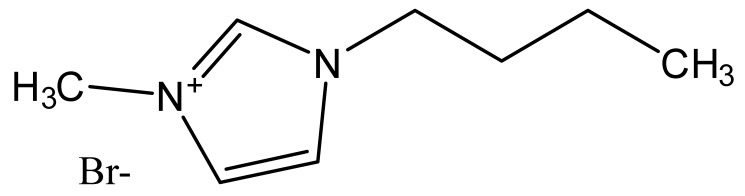
1-Butyl-3-methylimidazolium methanesulfonate	BMIMMeSO_3_	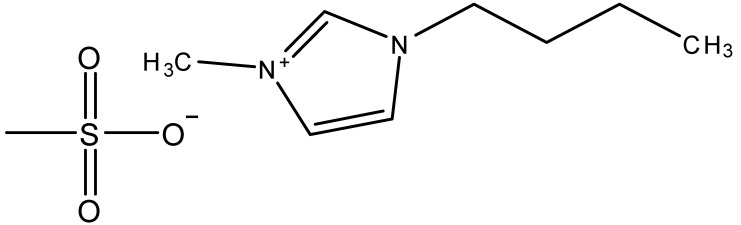
1-Ethyl-3-methylimidazolium diethyl phosphate	EMIMDep	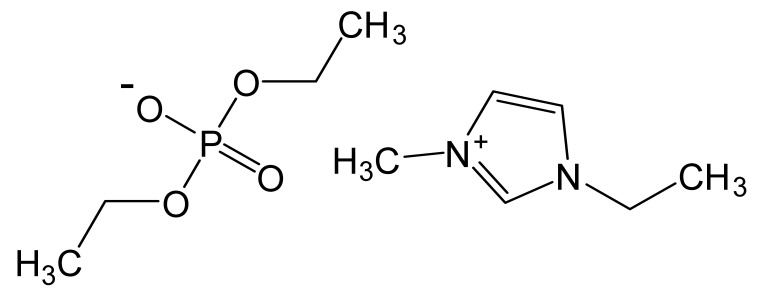
2-Hydroxyethylammonium formate	[NH_3_(CH_2_CH_2_OH)][OFo]	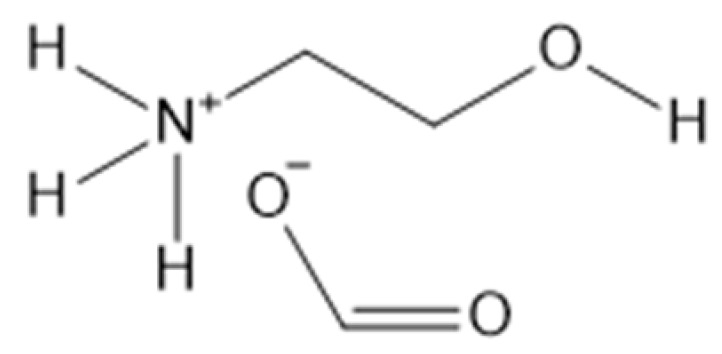
2-Hydroxyethylammonium acetate	[NH_3_(CH_2_CH_2_OH)][OAc]	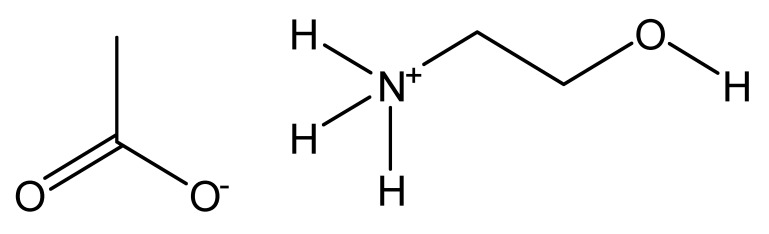
1-Ethyl-3-methylimidazolium hexafluorophosphate	[C_2_MIM][PF_6_]	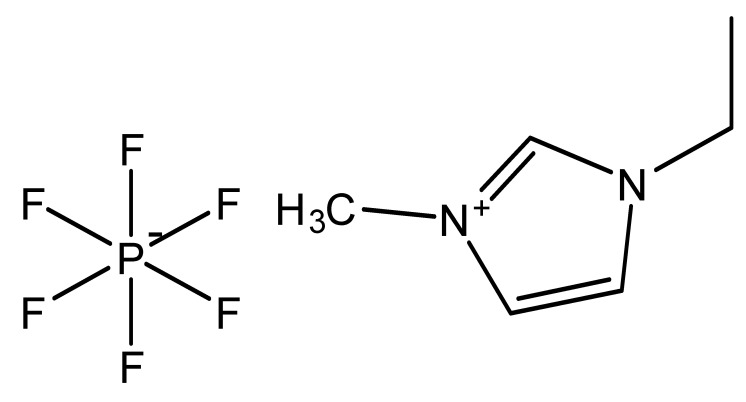
1-Hexyl-3-methylimidazolium hexafluorophosphate	[C_6_MIM][PF_6_]	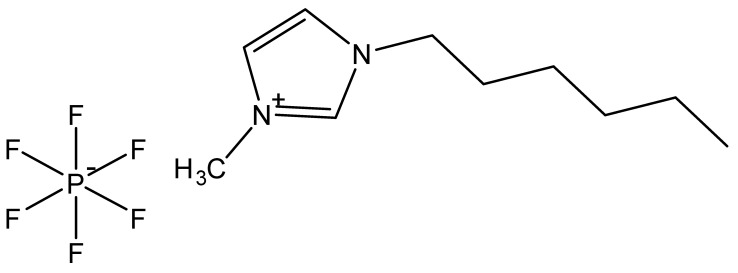
1-Ethyl-3-methylimidazolium trifluoromethanesulfonate	[C_2_MIM][Tf]	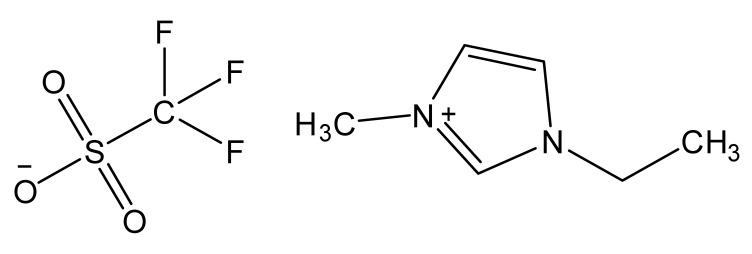
1-Butyl-1-methylpyrrolidinium bis(trifluoromethylsulfonyl)imide	[BMP][NTf_2_]	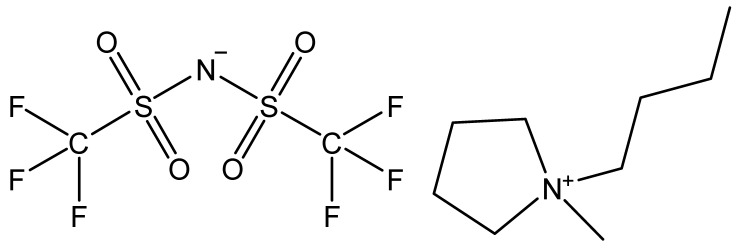
Triethylsulfonium bis(trifluoromethylsulfonyl)imide	[TES][NTf_2_]